# Improvement of Andean Blueberries Postharvest Preservation Using Carvacrol/Alginate-Edible Coatings

**DOI:** 10.3390/polym12102352

**Published:** 2020-10-14

**Authors:** Carolina Medina-Jaramillo, Carmen Quintero-Pimiento, Darío Díaz-Díaz, Silvia Goyanes, Alex López-Córdoba

**Affiliations:** 1 Facultad Seccional Duitama, Escuela de Administración de Empresas Agropecuarias, Universidad Pedagógica y Tecnológica de Colombia, Carrera 18 con Calle 22, Duitama 150461, Boyacá, Colombia; cmedina@df.uba.ar (C.M.-J.); rosaquintero885@gmail.com (C.Q.-P.); 2 Laboratorio de Polímeros y Materiales Compuestos (LP&MC), Departamento de Física, Instituto de Física de Buenos Aires (IFIBA-CONICET), Facultad de Ciencias Exactas y Naturales, Universidad de Buenos Aires, Buenos Aires C1428, Argentina; edgar3d300@hotmail.com (D.D.-D.); goyanes@df.uba.ar (S.G.)

**Keywords:** biopolymers, food preservation, agro-industry, berries, edible films

## Abstract

Edible coatings are attractive strategies for blueberries postharvest preservation. In this work, carvacrol/alginate coatings were developed for application on Andean blueberries. Coating formulations were prepared based on blends of sodium alginate (2% *w*/*v*), carvacrol (0%, 0.03%, 0.06% or 0.09%), glycerol, and water and applied to the fruits by dip-coating. Then, the fruits were immersed in a calcium batch to induce a crosslink reaction. Changes in the physicochemical and microbiological characteristics of the blueberries were monitored during 21 days of storage at 4 °C. Coated blueberries were better preserved throughout the 21 days of storage because of their lower respiration rate and water loss, in comparison with the uncoated ones. Besides, the coatings enhanced the appearance and the gloss of the fruits. Control fruits showed a significant decrease in the firmness, while, in the coated fruits, this critical postharvest quality was preserved during the entire storage. Coating formulations with 0.09% of carvacrol was the most effective in preventing mesophilic aerobic bacteria and molds/yeasts growth on the fruits during the storage. Edible carvacrol/alginate coatings can be considered as a useful alternative to complement the benefits of refrigerated storage by delaying post-harvest spoilage of Andean blueberries.

## 1. Introduction

Natural occurrent polymers (e.g., polysaccharides, proteins, and lipids) are appealing materials for many foods, pharmaceutical, biomedical and cosmetic applications because are abundant, biocompatible, non-toxic, and cheap. Between the wide variety of biopolymer applications, the development of edible coatings for food preservation has proved to be an excellent alternative to reduce food loss and waste and to improve food security [1]. 

Edible coatings are defined as food-grade blends of film-forming polymers plus solvents (generally water and/or alcohol) and other additives (e.g., plasticizers, antioxidants, antimicrobials, among others), which, when applied to a food’s surface by dipping or spraying, and then dried, produce an adherent, protective, decorative, and/or functional thin solid films [2]. Several studies show that the application of edible coatings in foods offer significant advantages because these improve their physical strength, decrease the gas exchange between the food and the environment, reduce water and aroma loss, delay color changes, and enhance the visual appearance of food product surfaces. Besides, coating with extra functionalities has been formulated thought the addition of antibacterial, antifungal, and antiviral agents that yield an outer surface capable of inhibiting the growth of foodborne pathogens and spoilage microorganisms [3,4]. 

Most of the commercially available edible coatings for fresh fruits and vegetables are based on waxes. However, it has been stated that consumers prefer unwaxed foods because some do not know why wax is used and link this material with health and environmental concerns [5]. Therefore, several investigations have been made to develop new edible coatings based on odorless, tasteless, and colorless natural polymers such as starch, chitosan, pectin, guar gum, and alginate, which do not exhibit adverse effects on the food sensory quality [6].

Alginates are a family of linear binary copolymers of (1–4)-linked β-d-mannuronic acid (M) and α-l-guluronic acid (G) monomers, which are naturally present in brown seaweed cell walls [7]. These are widely used in various industries (e.g., textile, agri-food, paper, cosmetic, biomedical, and pharmaceutical) as gelling, stabilizing, and thickening agents. Alginates can be turned insoluble through the cross-link with divalent or polyvalent cations such as Ca^2+^. This approach gives alginate very rigid conformation and a stable structure unlike the alginate with monovalent cations [8].

Alginates are an appealing raw material for the development of edible coatings because of their good film-forming properties and low permeability for fats, oils and, oxygen [9,10]. Moreover, alginates gels have temperature-dependent swelling behavior and this allows the controlled release of bioactive compounds [8]. Several studies show that alginate coatings have been highly effective in the preservation of several fruits and vegetables including blueberry, tomato, peach, sweet cherry, pineapples, and plums, among others [11,12,13,14]. In particular, alginate coatings supplemented with natural preservatives like essential oils and phenolic compounds have proven to be effective in inhibiting the growth of bacteria and fungi [9].

Carvacrol (5-isopropyl-2-methylphenol) is a bioactive isomeric monoterpenoid constituent of essential oils from thyme and oregano herbaceous plants (*Thymus vulgaris* L. and *Origanum vulgare* L., Lamiaceae family) [15], which is highly recognized by its potent antibacterial activity against both Gram-positive and Gram-negative bacteria [16,17]. It is considered generally recognized as safe (GRAS) for consumption by the U.S. Food and Drug Administration and is currently used in the food industry as a flavoring agent and in active food packaging applications [16]. 

There are few studies that deal with the effect of carvacrol-containing edible coatings on the preservation of fresh and minimally processed fruit and vegetables. Some works have been carried out about the development of carvacrol/biopolymer coating such as chitosan, starch, and zein [17,18,19]. Meanwhile, the development of alginate-based coatings with carvacrol has been few explored. Zapata et al. studied the effect of coating based on sodium alginate and their blends with essential oils (carvacrol, thymol, and eugenol) on the postharvest quality of sweet cherry fruits finding that coatings were able to reduce the water loss and the respiration rate and to delay color changes and fruit softening [10]. Peretto et al. have performed different studies dealing with the development of alginate coating with carvacrol and methyl cinnamate for strawberry preservation [20,21,22]. They reported that these coatings caused a significant reduction in visible decay and delay in the microbial spoilage of strawberries. Moreover, coated strawberries showed lower weight loss and higher fruit firmness than uncoated ones.

The Andean blueberry (*Vaccinium meridionale* Swartz) is a delicatessen fruit with high economic value and several health benefits, which grows in the Andean region of South America at 2300–3300 m above sea level (m.a.s.l.). This fruit is rich in bioactive compounds such as anthocyanins, flavonoids, and phenolic acids which have been identified as responsible for its antioxidant, cardioprotective, antiproliferative, and anti-inflammatory properties [23,24]. However, the Andean blueberry is a perishable fruit that decays rapidly during postharvest exhibiting weight loss, softening, color changes, and microbial growth. Therefore, there is a need to overcome these difficulties through the application of safe and environmentally friendly postharvest technologies for its preservation such as edible coatings. In our previous study, the effect of coatings based on starches and alginate/nanocellulose blends on the postharvest preservation of Andean blueberries was reported for the first time [6,25]. It was found that alginate coatings exhibited better performance on the weight loss reduction and the fruit firmness, in comparison with starch ones. In the current work, novel antimicrobial alginate edible coatings were developed and applied to fresh Andean blueberries in order to preserve their quality during postharvest storage. Coated and uncoated fruits were monitored during 21 days of refrigerated storage in terms of their pH, titratable acidity (%), soluble solids content, respiration rate, color, firmness, weight loss, phenolic compounds content, fungal decay, and microbial growth. To the best of our knowledge, this is the first report of the development of antimicrobial alginate-edible coatings with carvacrol for postharvest preservation of wild Andean blueberries.

## 2. Materials and Methods 

### 2.1. Materials

Andean blueberries (*Vaccinium meridionale* Swart) maturity stage 5 (100% dark purple) were obtained in Ráquira (Boyacá, Colombia) at 2150 m.a.s.l. The berries were examined previous to its use to separate fruits with physical, mechanical, or microbial damages. The fruits were washed and disinfected with a 100 mg L^−1^ chlorine solution. 

Carvacrol (98% purity), sodium hydroxide, and calcium chloride were purchased from Sigma-Aldrich (St. Louis, MO, USA). Sodium alginate was kindly donated by Saporiti (Buenos Aires, Argentina). Glycerol was purchased from J. T. Baker (Phillipsburg, NJ, USA) and Tween 80 was purchased from Loba Chemie (Mumbai, India). Folin-Ciocalteu reagement was purchased from Panreac (Barcelona, Spain) and gallic acid was purchased from Merck (Darmstadt, Germany). All chemicals used were of analytical grade.

### 2.2. Preparation of Coating Solutions

Coating solutions with and without carvacrol (CVR) were prepared as described in a previous work [25]. Alginate solutions were made by dissolving sodium alginate powder (2% *w*/*v*) in distilled water while heating at 70 °C under constant stirring until the mixture became clear. After cooling, glycerol (30% *w*/*v* dry weight basis) was added as a plasticizer to the sodium alginate solution and stirred for 15 min. For the preparation of alginate/CVR formulations, carvacrol was added to the alginate/glycerol blends at concentrations of 0.03%; 0.06% and 0.09% *w*/*v*, based on the studies of antimicrobial activity in vitro reported by Santos et al. [17]. Tween 80 was added to all alginate/CVR blends in amounts proportional to CVR (0.003%; 0.006% and 0.009% *w*/*v*, respectively) to assist dispersion. All mixtures were homogenized at 20,000 rpm for 3 min using an Ultra Turrax T25 homogenizer (IKA^®^ WERKE, Staufen, Germany), degassed using a vacuum pump, and cooled to room temperature before the application on the fruits.

### 2.3. Characterization of Edible Coatings

The surface solid density (SSD) was estimated as an indicator of the coating’s average thickness. Andean blueberries were covered as indicated in Section 2.3 and their surface solid density (g/m^2^) was estimated with the following equation [4,25]:

(1) $$\mathrm{SSD}=\left[\;M_{\mathrm{CA}}\cdot X_{s}/A_{s}\right],$$

where *M*_CA_ is the mass of the coating solution adhered to the fruit surface (g); *X*_S_ is the mass fraction of solid in the coating solution and A_S_ is the surface area of Andean blueberries (m^2^). The average sample surface area (*A*_s_) was estimated by considering each blueberry as a sphere. Samples were weighed before and after coating to determine the mass of the coating solution adhered to the fruit surface (*M*_CA_). The non-coated sample was used as a control. 

To characterize the coatings to water solubility, water vapor permeability and chemical conformation, films were performed by casting according to Medina-Jaramillo et al. [25]. Each coating solution was poured into polypropylene plates and dried at 50 °C for 24 h. Then, dried films were peeled from the plates, submerged in a gelling bath of calcium chloride solution (1% *w*/*v*) for 30 min, cleaned with distilled water, and air-dried at room temperature. All films were conditioned at room temperature into desiccators containing a supersaturated solution of sodium bromide (RH~57%) for 48 h before characterization studies. 

Water vapor permeability (WVP) tests were carried out at room temperature following the ASTM E96/ASTM E96M-16 method. Film samples were sealed over a circular opening of 4 × 10^−4^ m^2^ in a permeation cell, containing calcium chloride. Then, the cells were placed in desiccators conditioned with sodium chloride saturated solution (75% *RH*). Changes in the weight of the cell were recorded to the nearest 0.0001 g and plotted as a function of time, and the slope of each line was calculated by linear regression. *WVP* (g Pa^−1^ s^−1^ m^−1^) was calculated as follows:

*WVP* = (*WVTR*/*P*·*RH*) *d* (2)

where *WVTR* is the water vapor transmission rate calculated as the ratio between the slope of the straight line (g/s) and the cell area(m^2^); P is the saturation vapor pressure of water (Pa); *RH* is the relative humidity in the desiccator, and d is the film thickness (m).

The water solubility of the samples was assayed as reported in a previous work [26]. Film samples were weighed and submerged into a volume of distilled water (pH = 6.0) at room temperature (22–25 °C) for 24 h. Later, the water was removed, and the samples were dried at 1050 °C until constant weight. The mass loss during dissolving in water was calculated.

FTIR analysis was performed in a FT-IR 4100 spectrometer (Jasco, Hachioji, Tokyo, Japan) equipped with attenuated total reflectance (ATR) module. The samples were placed on the ATR accessory and then were analyzed under transmission mode, taking 64 scans per experiment with a resolution of 4 cm^−1^.

### 2.4. Application of Edible Coatings

A total mass of 7.5 kg of Andean blueberries was randomly divided into five groups (control, alginate, alginate/CVR 0.03%, alginate/CVR 0.06%, and alginate/CVR 0.09%), each group containing 1.5 kg of fruit. The blueberries were coated by immersion in the coating-forming blends for 90 s, drained of excess coating and submerged in a gelling bath of calcium chloride solution (1% *w*/*v*) for 30 min. Then, the coated fruits were cleaned with distilled water, and air-dried at room temperature. Control samples (without coating) were prepared by immersion in distilled water and air-dried at room temperature. These uncoated fruits were kept under the same storage conditions as the treated ones, for comparison [25].

### 2.5. Evaluation of Quality Attributes of Andean Blueberries along Storage 

The coated and uncoated Andean blueberries were packed in polyethylene terephthalate (PET) trays with perforated vents and stored for 21 days. Evaluations of quality attributes were performed at 0, 7, 14, and 21 days of refrigerated storage (4 °C and 90% *RH*). For every sampling time, three trays containing 125 g (~250 units) of Andean blueberries were prepared.

#### 2.5.1. Color Attributes

Color was measured using a tristimulus Minolta colorimeter (Konica-Minolta CR-10, Osaka, Japan) and was reported in CIELab parameters (L*, a* and b* values), where L* was used to denote lightness, a* redness and greenness, and b* yellowness and blueness. Hue angle values was calculated using the following equation:

(3) $$Hue\;angle=\;tan^{-1}(b^{*}/a^{*}),$$

#### 2.5.2. Respiration Rate

Respiration rate was measured as reported Medina-Jaramillo [25]. Approximately 120 g of Andean blueberries was placed for 30 min at 25 °C inside hermetically sealed 2 L flasks. Then, the CO_2_ concentration was determined using an infrared analyzer (LabQuest^®^2 Model LQ2-LE, Beaverton, OR, USA). The results were expressed in mg kg^−1^ s^−1^. 

#### 2.5.3. Weight Loss 

Weight loss of the Andean blueberries during storage was determined by weighing all fruit trays at the beginning of the storage and every day of analysis. The weight loss (% *W*) was calculated with the following equation:

(4) $$\%\;W=\;\left(\frac{m_{0}-m_{f}}{m_{0}}\right)\times \;100,$$

where *m*_f_ is the weight at each time and m_0_ the initial weight of each sample.

#### 2.5.4. Soluble Solids Content, pH, and Titratable Acidity (%)

The soluble solids content was measured in the fruit juice using an Atago refractometer model PR 101 (Atago CO., Tokyo, Japan) and expressed as Brix (AOAC 932.12). Fruit samples were crushed using a blender and filtered through filter paper to obtain the fruit juice. 

The pH of the fruit samples was assessed using a digital pH meter (Oakton Instruments, Vernon Hills, IL, USA) (AOAC 981.12).

Titratable acidity (%) was determined by titration with 0.1 N NaOH up to pH 8.2, using 0.5 g of sample in 10 mL of distilled water (AOAC 942.15). The results were expressed as citric acid percentage. 

#### 2.5.5. Firmness Analysis

Firmness was determined using a digital Force Gauge PCE-FM200 (Southampton, UK) equipped with a 6 mm diameter stainless steel probe. Firmness was defined as the maximum force (N) to disrupt the tissue at the penetration time used (5 s) [27]. The results were expressed as an average of at least five measurements.

#### 2.5.6. Total Polyphenols Content

For the determination of the total polyphenols content, aqueous extracts of the fruits were prepared. Blends of crushed fruits (3 g) and distilled water (100 mL) were placed in a thermostatic bath at 50 °C for 30 min. Once obtained, the extracts were cooled and filtered. 

Total polyphenols content was determined by the Folin–Ciocalteu method [28,29]. Briefly, 400 μL of fruit aqueous extract was mixed with 2 mL of Folin–Ciocalteu reagent (1:10 diluted). Then, 1.6 mL of sodium carbonate (7% *w*/*v*) was added to each sample. After 30 min, the absorbance was measured at 760 nm using a spectrophotometer (X-ma 1200 Human Corporation, Loughborough, UK). The results were expressed as gallic acid equivalents (GAE) per gram of fresh fruit.

#### 2.5.7. Microbiological Analysis 

The determination of aerobic mesophilic bacteria was done according to ISO 4833-1: 2013 standard [30]. To count molds and yeasts, the assay was done according to ISO 21527-1,2: 2008 standard [31]. Briefly, a known amount of each sample was aseptically taken and homogenized with sterile peptone water. Then, decimal dilutions of homogenate and sterile peptone water were performed. For the determination of aerobic mesophilic bacteria, 100 μL of each dilution were plated onto plate count agar and the plates were incubated for 2 days at 35 °C. For molds and yeasts, 100 μL of the dilutions were spread onto potato dextrose agar and the plates were incubated for 5 days at 25 °C. After incubation, colonies were counted and the results were expressed in log colony-forming units per gram (log_10_ CFU. g^−1^).

#### 2.5.8. Fruit Decay Evaluation

The external appearance of each fruit and the presence of macroscopic fungal growth were visually evaluated during storage period. Fruits which showed surface mycelial development or bacterial lesions were considered as decayed. Three different tests were performed each in duplicate. Results were expressed as the percentage of decayed fruit.

### 2.6. Sensory Evaluation

Sensory quality of control and coated Andean blueberries with alginate and alginate/CVR 0.09% was evaluated using a hedonic test. Ninety-eight people, aged between 18 and 60 years old, were randomly recruited from the city of Duitama (Colombia). Fruit samples were prepared 1 day before the day of the sensory study, stored at a 4 °C refrigerator after processing, and then warmed up to ambient temperature at the day of test before serving to consumers. Fruit samples were placed in plastic cups labeled with 3-digit random numbers. The consumers were asked to evaluate the overall acceptability, appearance, color, taste, texture, and odor using a 9-point hedonic scale (1 = dislike extremely, 5 = neither like nor dislike, and 9 = like extremely).

### 2.7. Statistical Analysis

The statistical analysis was performed using Minitab v. 16 statistical software (State College, PA, USA). Analysis of variance (ANOVA) and Tukey’s pairwise comparisons were carried out using a level of 95% confidence. The experiments were performed at least in triplicate, and the data were reported as mean ± standard deviation.

## 3. Results and Discussion

### 3.1. Coating Characterization

Thickness plays an important role in the coating functionality because it is related to the transport properties of the material [32]. It has been reported that the incorporation of additives in the coatings could affect their thickness [32]. In the current work, the surface solid density (*SSD*) was estimated as an indicator of the coating’s average thickness. Alginate coatings without carvacrol showed *SSD* values of 3.7 g/m^2^; while all coatings with carvacrol showed *SSD* values of 3.3 g/m^2^ (i.e., lower thickness than alginate coating), regardless of the carvacrol concentration used. This slight *SSD* decrease caused by the carvacrol addition could suggest a good homogeneity and compatibility of the coatings. *SSD* values between 1.0 and 1.8 g·m^−2^ have been reported for blueberries (*Vaccinium corymbosum*) covered with carrageenan coatings [4]. 

The low concentrations of carvacrol added (0.03%, 0.06%, and 0.09%) did not cause changes in the water vapor barrier properties of the alginate coatings. In this sense, all systems showed similar water vapor permeabilities (5 × 10^−9^ g s^−1^ m^−1^ Pa^−1^). Rojas-Grau, et al. [33] when worked with alginate–apple puree films containing higher concentration (0.1% and 0.5%) of plant essential oils (oregano, lemongrass, and cinnamon) and oil compounds (carvacrol, citral, and cinnamaldehyde) reported that these did not modify the WVP of the films. Besides, the different concentrations of carvacrol used did not have a significant effect (*p* < 0.05) on the water solubility of the coatings and all systems showed high-water resistance (water solubility below 1%). Tapia et al. stated that alginate films have a resistance to being dissolved in water and, therefore, have the potential for coating high moisture fresh foods [34].

**Figure 1:**
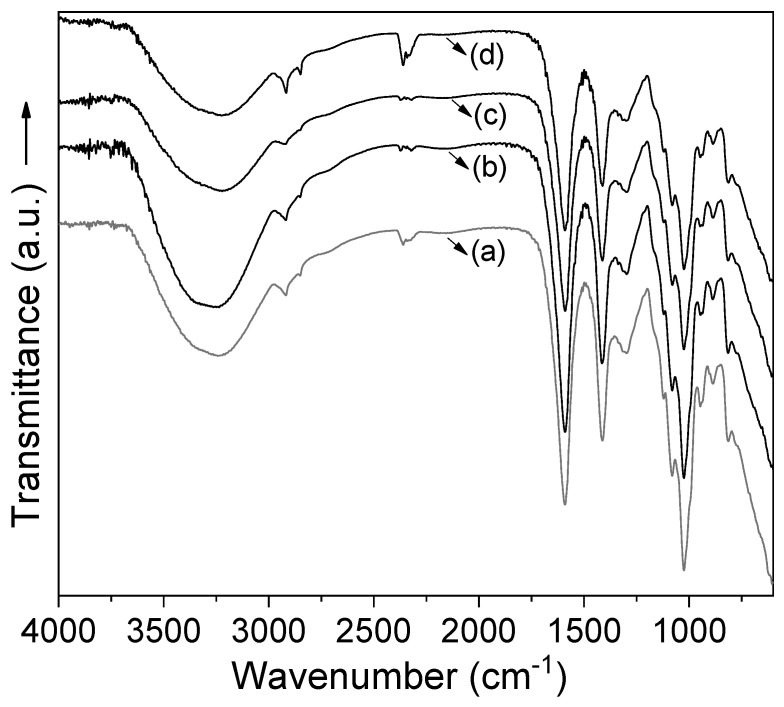
IR spectra of calcium alginate coatings without and with carvacrol (CVR): (**a**) alginate, (**b**) alginate/CVR 0.03%, (**c**) alginate/CVR 0.06% and (**d**) alginate/CVR 0.09%.

Fourier transform infrared spectrometry of all coatings was performed to study the interactions between carvacrol and alginate (Figure 1). IR spectra of alginate and alginate/carvacrol samples showed characteristic bands of calcium alginate at 3248 cm^−1^ (O–H stretching), 1591 cm^−1^ (asymmetric stretching vibration of C–O bond of COO– group) and 1413 cm^−1^ (symmetric stretching vibration of C–O in COO– group). The peak at 1028 cm^−1^ corresponds to the antisymmetric stretch of C–O–C and the peak at 818 cm^−1^ is characteristic of mannuronic acid residues [35]. Besides, in the alginate/CVR coatings the carvacrol characteristics signals were probably overlapped by other spectrum signals. This behavior was probably due to both the very low molar ratio of carvacrol in the coatings and the lack of covalent bonds between carvacrol and alginate.

### 3.2. Effect of Edible Coatings on Andean Blueberries

**Figure 2:**
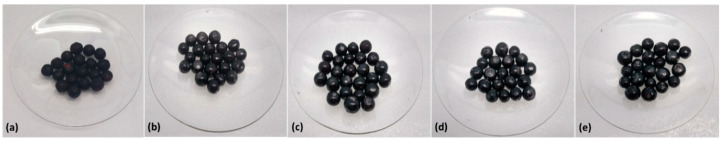
Images of Andean blueberries without and with alginate and alginate/carvacrol coatings: (**a**) control; (**b**) alginate; (**c**) alginate/CVR 0.03%; (**d**) alginate/CVR 0.06% and (**e**) alginate/CVR 0.09%.

**Figure 3:**
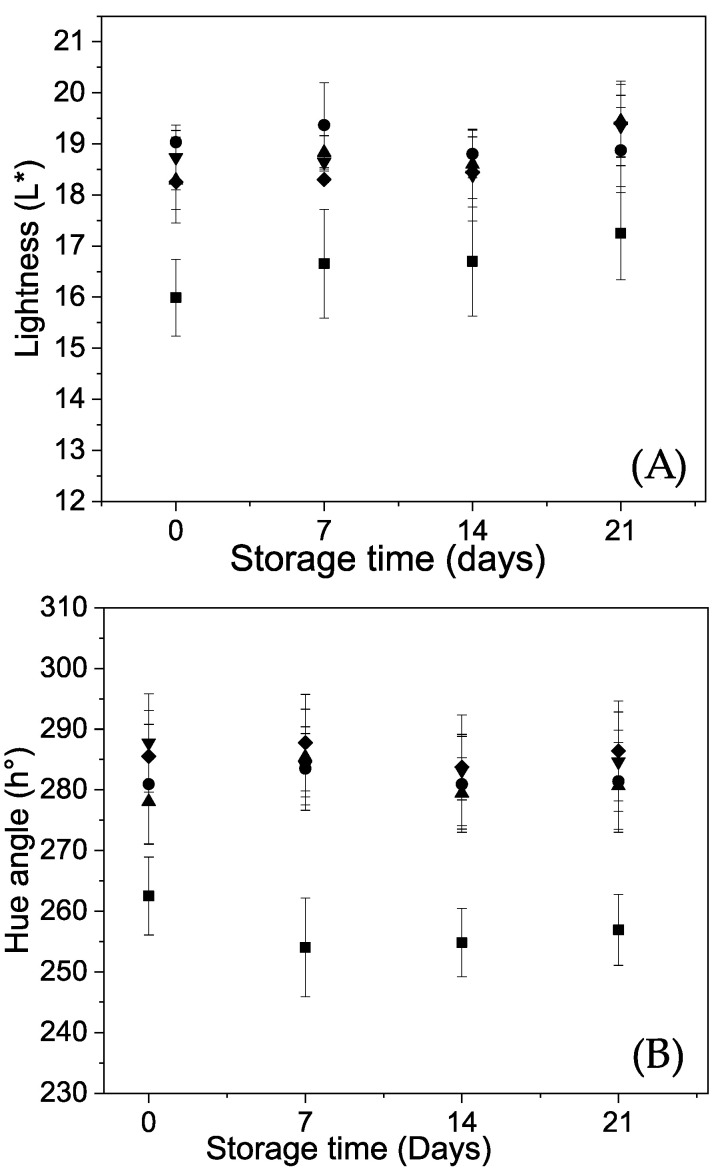
Behavior of the Andean blueberry lightness (**A**) and hue angle (**B**) during storage. Control (■), alginate (●), alginate/CVR 0.03% (▲), alginate/CVR 0.06% (▼), alginate/CVR 0.09% (♦).

Images of Andean blueberries with and without coatings are shown in Figure 2 and the behavior of the color attributes of the fruits during storage are shown in Figure 3. 

Uncoated fruits showed lower lightness (L*) (i.e., a darker color) than coated ones; while not statically significant differences were found between the lightness obtained for the fruits coated with alginate and alginate-carvacrol (alginate/CVR 0.03%; alginate/CVR 0.06% and alginate/CVR 0.09%) during the storage (Figure 3A). This appearance improvement could be due to that the smoother surface of the coated fruits caused a greater visible light reflection [6,36]. Similar behaviors have been reported in strawberries coated with chitosan-oleic acid [36] and with alginate-carvacrol [20]. At the beginning of the storage, Andean blueberries have a characteristic dark purple color with a hue angle of 263 for the uncoated fruits (Figure 3B). After 7 days of storage, these fruits showed a slight decrease in the hue angle (254), and then maintaining this parameter—almost unchanged—until the end of storage (Figure 3B). Zapata et al. [37] observed a reduction in the hue angle in sweet cherries during the refrigerated storage and it was attributed to the advance of the ripening process of the fruits. All coated Andean blueberries (alginate; alginate/CVR 0.03%; alginate CVR 0.06% and alginate/CVR 0.09%) showed higher hue angles at the beginning and during the entire storage than the uncoated ones (Figure 3B). Besides, the hue angle of the coated fruits was maintained almost unchanged until the end of storage. Therefore, it can be stated that these coatings delayed the color changes of the fruits. The main color pigments in Andean blueberries are cyanidin and delphinidin glycosides [38]. Several authors have reported that during the storage, color changes could occur due to oxidations and/or condensation reactions of anthocyanins with other phenolic compounds [39,40]. In coated fruits, it has been postulated that color changes could be delayed because coatings cause oxygen depression, retard the anthocyanin synthesis associated to the postharvest ripening process and prevent the release of cellular fluids containing enzymes and substrates of browning [12,41].

**Figure 4:**
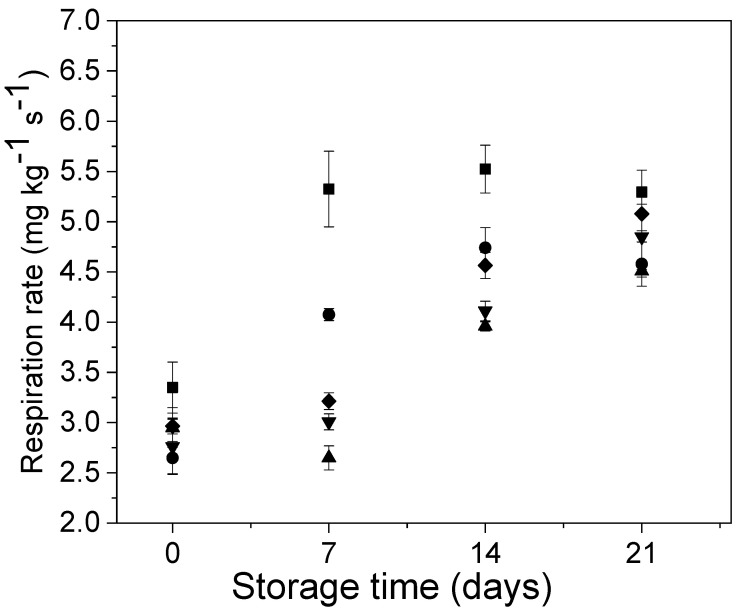
Behavior of the Andean blueberry respiration rate during storage. Control (■), alginate (●), alginate/CVR 0.03% (▲), alginate/CVR 0.06% (▼), alginate/CVR 0.09% (♦).

The changes in the respiration rate of Andean blueberries with and without edible coatings during storage are shown in Figure 4. At the beginning of the assay, the respiration rate of the coated samples was slightly lower than the uncoated samples. After 7 days of storage, the fruits uncoated and coated with alginate without carvacrol showed increases of 70% and 50% in their respiration rate, respectively; while, in the fruits coated with alginate/CVR 0.03%, alginate/CVR 0.06% and alginate/CVR 0.09% the respiration rate was maintained almost unaltered. In the following 7 days, all samples showed an increase in their respiration rate. Besides, it was noted that from the day 7 of storage the respiration rate of fruits coated with alginate/CVR decreased in the following order: alginate/CVR 0.03% < alginate/CVR 0.06% < alginate/CVR 0.09%. At the end of the storage, the Andean blueberries coated with alginate and alginate/CVR 0.03% showed a lower respiration rate than the uncoated fruits, and no significant differences were found between those and alginate/CVR 0.06 and alginate/CVR 0.09 samples (*p* > 0.05). The decrease in the respiration rate of fruits and vegetables caused by the application of edible coatings has been reported by several studies [42]. It has been stated that edible coatings based on carbohydrates, such as alginate, increase the skin resistance to gas diffusion because they produce a tightly packed, ordered hydrogen-bonded network structure which leads to blocking the pores on the fruit surface and causes a modified internal atmosphere of relatively high CO_2_ and low O_2_ which decreases the intensity of physiological processes [10,42,43]. This effect has been improved with the supplementation of the edible coatings with essential oils such as the carvacrol because these additives improve the coating adherence on the fruit surface [10]. However, during storage, component volatilization could occur and it could cause a reduction in the gas barrier properties of the edible coatings. This could have been the reason why the respiration rate of the fruits coated with alginate/CVR 0.06 and alginate/CVR 0.09 increased towards the end of storage.

Table 1 shows the behavior of the weight loss of Andean blueberries with and without edible coatings. All systems showed an increase in their weight loss until the end of the storage. However, this parameter was significantly delayed for the coated fruits with and without carvacrol (alginate, alginate/CVR 0.03%, alginate/CVR 0.06% and alginate/CVR 0.09%). At 21-days of storage, the fruit coated with alginate showed a decrease in the water loss of around 36%, with respect to the uncoated fruits; while, the fruits with alginate/CVR showed a decrease of around 52%, regardless of the carvacrol concentration. The greater water loss of uncoated fruits was probably due to an increase in the fruit metabolic activity which led to greater transpiration and respiration rate [37]. While, in the other fruits, the water vapor barrier provided by the alginate coatings delayed the water loss, mainly when carvacrol which is a hydrophobic terpene was added. These findings were consistent with previous studies in which the application of alginate and alginate-carvacrol coatings minimize the water loss from fresh berry fruits such as strawberries and cherries [20,44]. 

Concerning soluble solids content, titratable acidity and pH, the fruits with and without the coating did not show significant changes in these parameters thought the storage obtaining values around 16.5 °Brix, 2.2% and 2.5, respectively (i.e., edible coatings did not affect negatively these fruits characteristics). Similar results have been reported by other authors for Andean blueberries [24]. 

**Figure 5:**
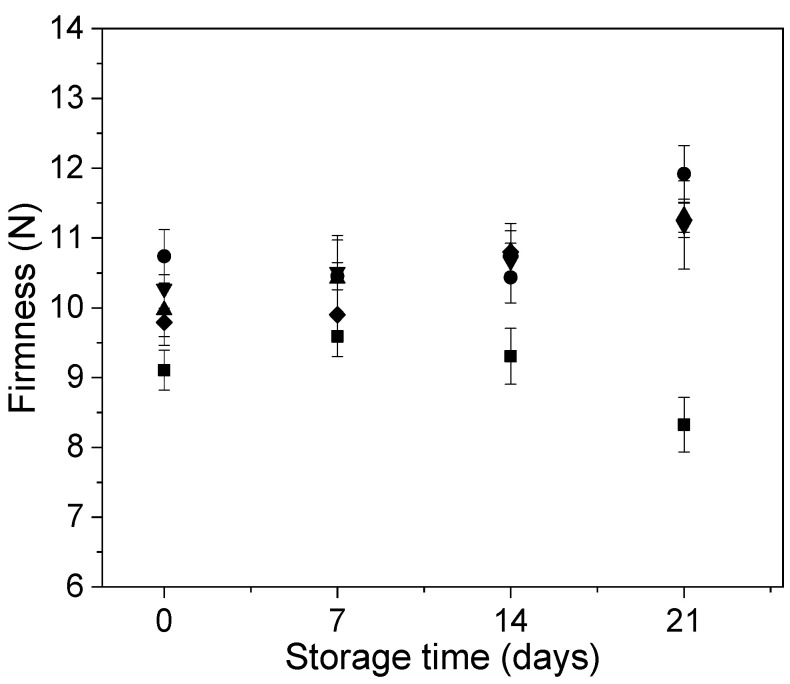
Behavior of the Andean blueberry firmness during storage. Control (■), alginate (●), alginate/CVR 0.03% (▲), alginate/CVR 0.06% (▼), alginate/CVR 0.09% (♦).

The firmness behavior of Andean blueberries with and without edible coatings during storage is shown in Figure 5. The application of the coatings generated a slight increase in the firmness of the fruits compared to the control ones during the entire refrigerated storage. Uncoated fruit showed similar firmness until day 7 of storage. After this period, these fruits showed a decrease in this parameter probably due to the softening caused by the increase in their metabolic and enzymatic activity [45]. In the case of the coated fruits with and without carvacrol, the softening was delayed and the fruit maintained their firmness from the initial time until the end of the storage. This behavior agrees with the lower values of respiration rate (i.e., lower metabolic activity) observed for the coated samples (Figure 4). It has been reported that both the effective gas barrier properties of alginate edible coatings and the use calcium chloride for polymers crosslinking, contribute to the reduction in metabolic activity of fruits and helped to maintain better fruit firmness [20,46]. Chiabandro et al. [45] reported that the use of alginate edible coating applied on blueberries showed beneficial effects on firmness retention during 45 days of storage and this was attributed to the retarded degradation of components responsible for structural rigidity of the fruit such as insoluble pectin and proto-pectin. 

**Figure 6:**
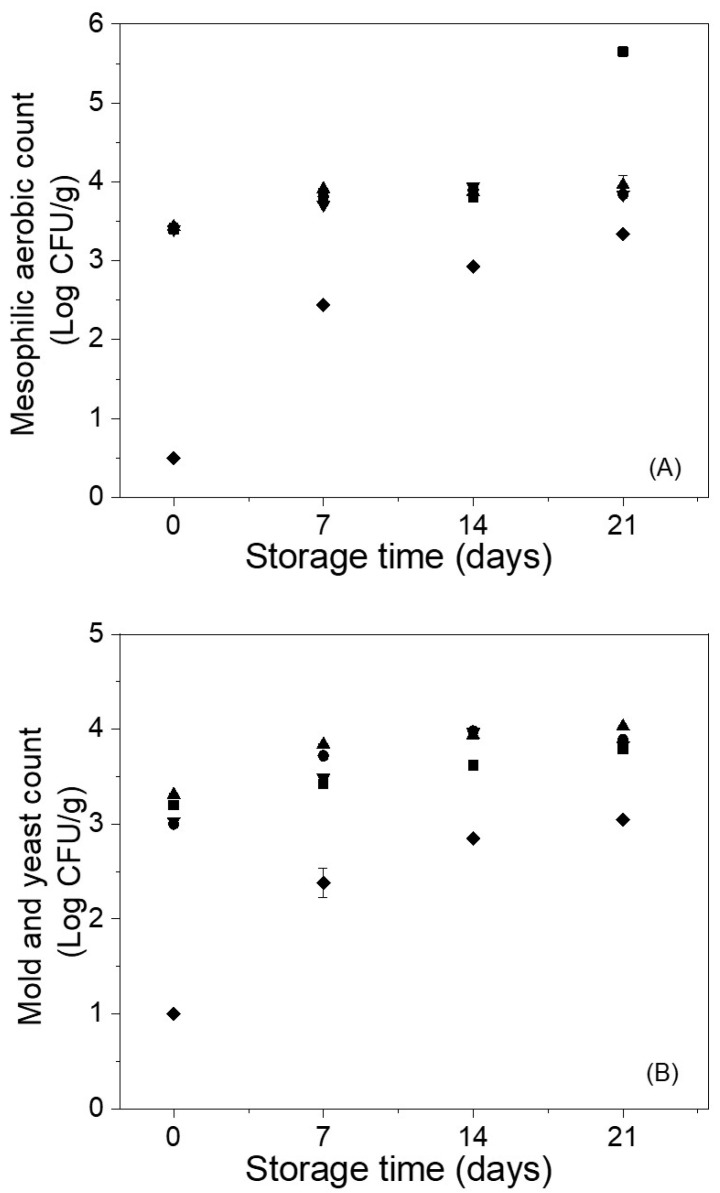
Behavior of the mesophilic aerobic (**A**) and molds/yeasts (**B**) grown on Andean blueberries during storage. Control (■), alginate (●), alginate/CVR 0.03% (▲), alginate/CVR 0.06% (▼), alginate/CVR 0.09% (♦).

The behavior of the mesophilic aerobic and molds/yeasts grown on Andean blueberries with and without alginate or alginate/carvacrol coatings during refrigerated storage is shown in Figure 6. In the first 14 days of storage, uncoated fruits showed mesophilic aerobic bacteria count ranging from 3.4 to 3.8 log CFU g^−1^ (Figure 6A). After this time, the total colony counts of these samples increased by about 1.8 log cycles. The coated fruits with alginate, alginate/CVR 0.03%, and alginate/CVR 0.06% showed a more similar mesophilic aerobic bacteria count than control samples during the first 14 days of the assay (Figure 6A). At the end of storage, these coated samples maintained their total colony counts unaltered (~3.8 log CFU g^−1^). The application of alginate coatings with carvacrol at 0.09% (alginate/CVR 0.09%) had a marked effect in reducing the population of mesophilic aerobic bacteria as compared to the other samples. At initial time, the fruits coated with alginate/CVR 0.09% showed mesophilic aerobic bacteria count <2 log CFU g^−1^ (Figure 6A). Then, a gradual increase in the microbial count was observed reaching values as high as 3.3 log CFU g^−1^ (i.e., the effectiveness of carvacrol decrease during the storage time). It has previously been reported that the use of alginate coating did not exert significant effects on the mesophilic aerobic bacteria count and that the supplementation of these coatings with carvacrol is a useful strategy to render antimicrobial activity [47]. Several mechanisms have been proposed for explaining the antibacterial activity of carvacrol including the disruption of bacterial membrane leading to bacterial lysis and leakage of intracellular contents resulting in death [16]. Other proposed mechanisms of antibacterial action include the inhibition of efflux pumps, prevention in the formation and disruption of preformed biofilms, inhibition of bacterial motility, and inhibition of membrane ATPases [16]. Sun et.al. [18], when studying the effect of a chitosan coating with carvacrol on blueberries, found that the coatings maintained fruit firmness and reduced microbial growth on the fresh fruit during storage mainly when carvacrol dosages higher than 0.1% were used. 

The application of edible coating based on alginate, alginate/CVR 0.03% and, alginate/CVR 0.06 did not exert significant effects on the yeasts and molds count of Andean blueberries. These samples showed similar yeast/mold levels than the uncoated fruits along the storage (3.0–4.0 log CFU g^−1^) (Figure 6B). The fruits coated with alginate/CVR 0.09% showed yeasts and molds count <2 log CFU g^−1^ at the beginning of storage (Figure 6B). Then, the effectiveness of carvacrol decreases, and a gradual increase in the yeast and mold levels of these samples was observed reaching values as high as 3.0 log CFU g^−1^. From the shelf life point of view, it has been reported that in non-thermal processed fruits the maximum acceptable yeast count is 6 log CFU g^−1^ [48]. In this sense, all samples can be considered as safe for consumption, and their shelf life was not limited by yeast count during the entire period of storage. 

**Figure 7:**
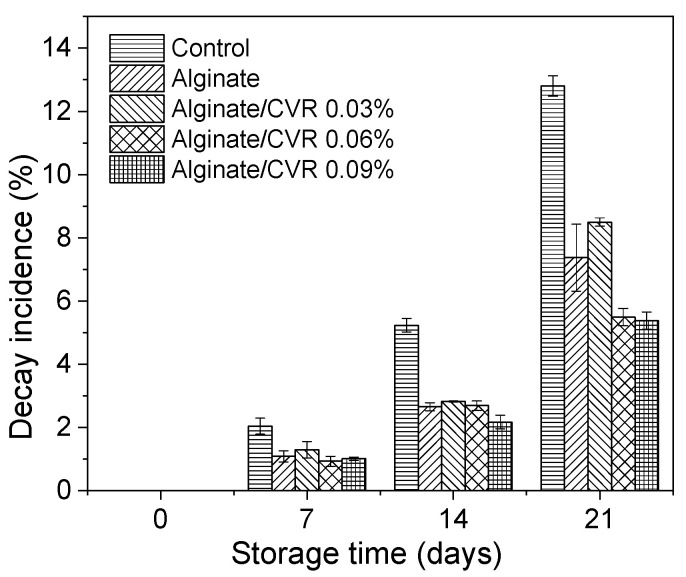
Changes in Andean blueberry decay incidence during storage.

Visual inspection is particularly important for bacterial rots and mold contamination and spoilage. It has been reported that yeasts and molds are considered the main spoilage agents due to the low pH of most fruits [47]. The decay of Andean blueberries with and without edible coatings was evaluated during storage (Figure 7). In general, all the fruits showed a gradual increase in the decay during the entire storage. However, the uncoated fruits showed a higher decay rate than the coated ones (alginate, alginate/CVR 0.03%, alginate/CVR 0.06%, alginate/CVR 0.09%). At end of the storage period, all coatings applied significantly reduced the apparition of decay signs in the Andean blueberries in comparison with the uncoated ones. This inhibitory effect was much more noted in the fruits coated with alginate/CVR 0.06% and alginate/CVR 0.09%. Similar behavior had reported several authors and it has been attributed to that coatings create a modified atmosphere on fruit surface that may inhibit microbial growth during postharvest storage, resulting in a lower amount of decayed fruits [47].

**Figure 8:**
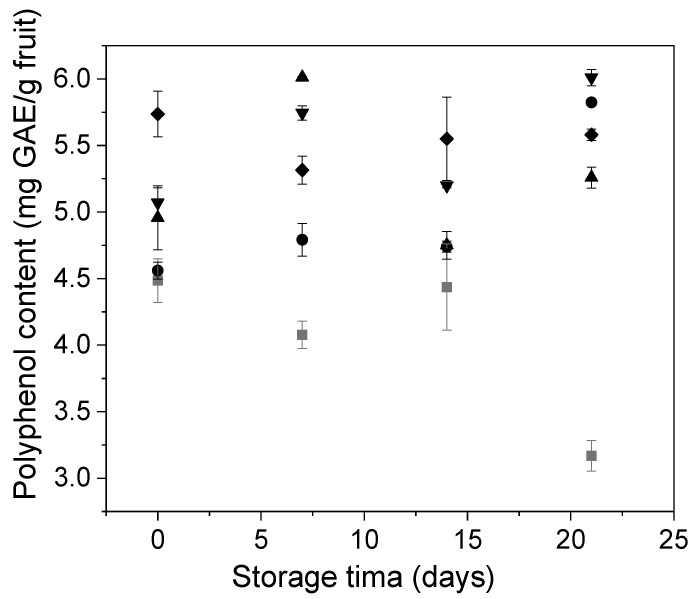
Changes in Andean blueberry polyphenol content during storage. Control (■), alginate (●), alginate/CVR 0.03% (▲), alginate/CVR 0.06% (▼), alginate/CVR 0.09% (♦).

The changes in Andean blueberry polyphenol content during storage are shown in Figure 8. At the initial timepoint, control and alginate coated fruits showed similar polyphenols content (~4.5 mg GAE/g) to those reported in the literature [23]. As expected, the fruits coated with alginate/carvacrol (alginate/CVR 0.03%, alginate/CVR 0.06%, and alginate/CVR 0.09%) showed higher polyphenol content than control and alginate coated fruits. This increase was attributed to that the presence of carvacrol, which is a monoterpenic phenol, contributed to the total polyphenol content of the fruit. Similarly, Yuan et al. [49] reported an increase in the polyphenol content and the antioxidant activity of chitosan films when they were combined with carvacrol. Along with the storage, the behavior of the polyphenol content of the fruits with and without edible coatings did not follow a common pattern and was different for each one. Uncoated fruits showed a significant decrease in their polyphenol content after 15 days of storage, while the coated samples showed similar or higher polyphenol concentrations compared to that at the initial time, indicating that the coatings were able to prevent polyphenol loss in Andean blueberries. Alvarez et al. [47] observed a similar behavior in the polyphenol concentration with the application of alginate and chitosan coatings enriched with dietary fibers on blueberries during 18 days of storage at 5 °C. The fluctuations in the polyphenol content may be related to the tendency of polyphenols to undergo polymerization reactions [50].

**Figure 9:**
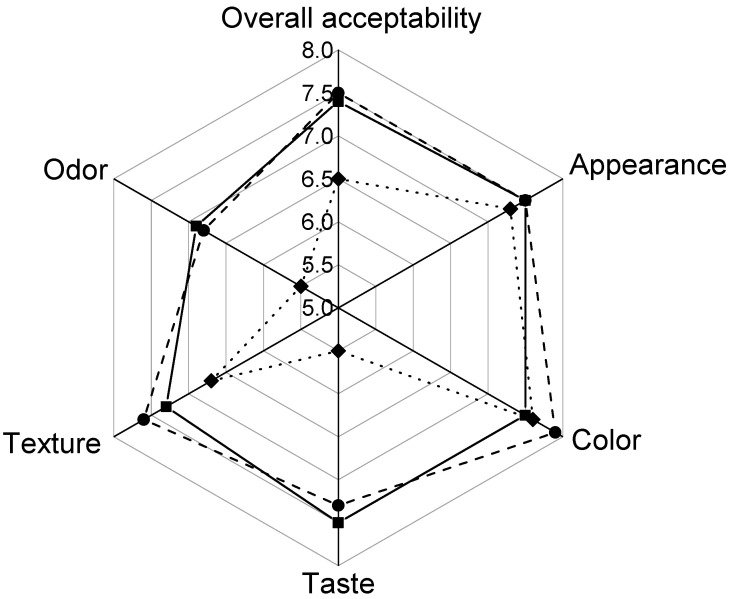
Radar chart representing mean scores of the sensory analyses for control and coated Andean blueberries. Control (■), alginate (●), alginate/CVR 0.09% (♦).

It has been reported that the incorporation of natural antimicrobial agents into edible coatings could change the original flavors of foods due to the strong flavors associated with them [46]. Therefore, the evaluation of the sensory characteristic of the new coated Andean blueberries is very important to make sure consumer acceptability is not compromised. Several authors have reported that samples can be considered acceptable when they received scores higher than or equal to five [51]. Scores below five indicated that the samples were disliked by the consumers [51]. Figure 9 shows the effect of the application of alginate coatings without and with carvacrol on the sensory attributes of Andean blueberries. All samples received scores higher than five indicating good consumer acceptability. It can be observed that the fruits coated with alginate showed similar sensory characteristics than uncoated ones indicating that the edible coating did not modify the sensory attributes of the fresh fruit. While the alginate/CVR 0.09% coatings had a significant effect on the overall acceptability, taste, texture, and odor of the fruits. Despite the low concentration of carvacrol used, some consumers detected a residual aromatic herbal taste which diminished the sensory acceptance of these samples (Figure 9). Concerning attributes of appearance and color, unimportant differences were found in comparison with the control fruits and those coated with alginate. 

## 4. Conclusions

Alginate coatings supplemented with carvacrol proved to be an effective postharvest treatment to prevent Andean blueberry water loss, softening and, microbial decay. Moreover, these coatings were useful for enhancing the polyphenol content of the fruits. These are important results since these attributes are closely related to consumer acceptance. The coating formulation containing 0.09% of carvacrol proved to be the most effective in improving the postharvest quality and delayed mesophilic bacteria and yeasts/molds grown during 21 days of refrigerated storage. Besides, these samples were ranked above the limit of consumer sensory acceptability. Therefore, the application of these edible coatings may be an interesting approach to preserve fresh fruit quality and to improve the microbiological safety of Andean blueberries during postharvest storage. More studies are necessary to validate the coating performance on Andean blueberries and other fruits at the industrial scale.

## Figures and Tables

**Table 1:** Behavior of the weight loss of Andean blueberries fruits without and with alginate or alginate/carvacrol coating during storage.

Weight Loss (%)					
Storage Time (Days)	Control	Alginate	Alg/CVR 0.03%	Alg/CVR 0.06%	Alg/CVR 0.09%
7	4.4 ± 0.2 ^a^	2.7 ± 0.5 ^b^	3.0 ± 0.9 ^b^	2.8 ± 0.6 ^b^	3.3 ± 0.5 ^b^
14	8.1 ± 1.6 ^a^	4.9 ± 0.8 ^b^	6.3 ± 1.1 ^b^	6.3 ± 0.7 ^b^	6.2 ± 0.4 ^b^
21	15.2 ± 1.0 ^a^	9.7 ± 2.0 ^b^	7.4 ± 1.2 ^b^	7.3 ± 0.6 ^b^	7.3 ± 0.4 ^b^

Different letters within the same row indicate statistically significant differences (*p* < 0.05).
